# Rescuing the Hidden Canine: A Case Report of Successful Surgical Exposure and Orthodontic Management

**DOI:** 10.7759/cureus.49888

**Published:** 2023-12-04

**Authors:** Lovely Bharti, Sunita S Shrivastav, Abhishek D Sanchla, Ranjit Kamble

**Affiliations:** 1 Orthodontics and Dentofacial Orthopaedics, Sharad Pawar Dental College and Hospital, Datta Meghe Institute of Higher Education & Research, Wardha, IND

**Keywords:** balanced functional occlusion, closed eruption, orthodontic treatment, interdisciplinary approach, canine impaction

## Abstract

Canine impaction, a recognized dental condition, particularly in the maxillary region, poses both functional and esthetic challenges. This case report explores the management of impacted maxillary canines coexisting with missing upper third molars in the same patient, showcasing the complexity of dental anomalies. Its multifaceted etiology includes complex eruption pathways and potential genetic factors. Addressing the impacted canine (upper left), particularly in the anterior region, is essential for oral health and aesthetics. Surgical-orthodontic techniques, guided eruption, and interdisciplinary collaboration have revolutionized management. This report emphasizes early diagnosis, personalized treatment, and the transformative potential of surgical exposure and orthodontic intervention in enhancing oral health, function, and aesthetics.

## Introduction

Canine impaction, a dental condition where a permanent canine tooth fails to erupt into its proper position within the dental arch, presents a challenging dilemma in the field of orthodontics [1]. This condition, although not uncommon, demands a multifaceted approach to its diagnosis, treatment planning, and resolution. In this case report, we present a unique and compelling clinical scenario that highlights the successful management of canine impaction through a carefully orchestrated combination of surgical exposure and orthodontic intervention. The significance of this case lies not only in its successful outcome but also in the broader context of the clinical challenges posed by canine impaction [2]. Impacted canines can have profound implications for oral health, aesthetics, and occlusal stability [3]. Early diagnosis and appropriate treatment are paramount to prevent potential complications, such as root resorption, cyst formation, or malocclusion [4]. This case report aims to contribute to the growing body of knowledge surrounding canine impaction management by offering a detailed account of the diagnostic process, the intricacies of surgical exposure, the nuances of orthodontic guidance, and the ultimate achievement of a favorable clinical outcome. It underscores the importance of a collaborative and multidisciplinary approach involving orthodontists, oral surgeons, and other dental specialists [5].

## Case presentation

**Figure 1:**
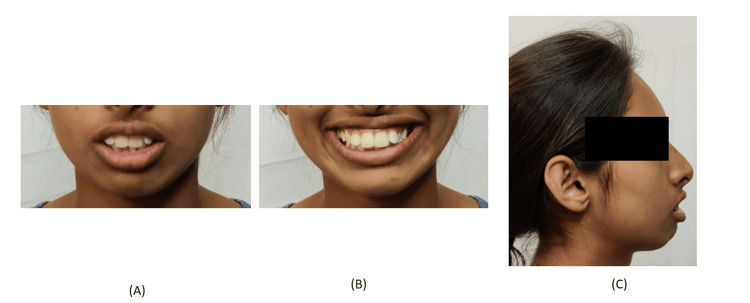
Pre-treatment extraoral photograph (A) Frontal, (B) smiling, and (C) profile

**Figure 2:**
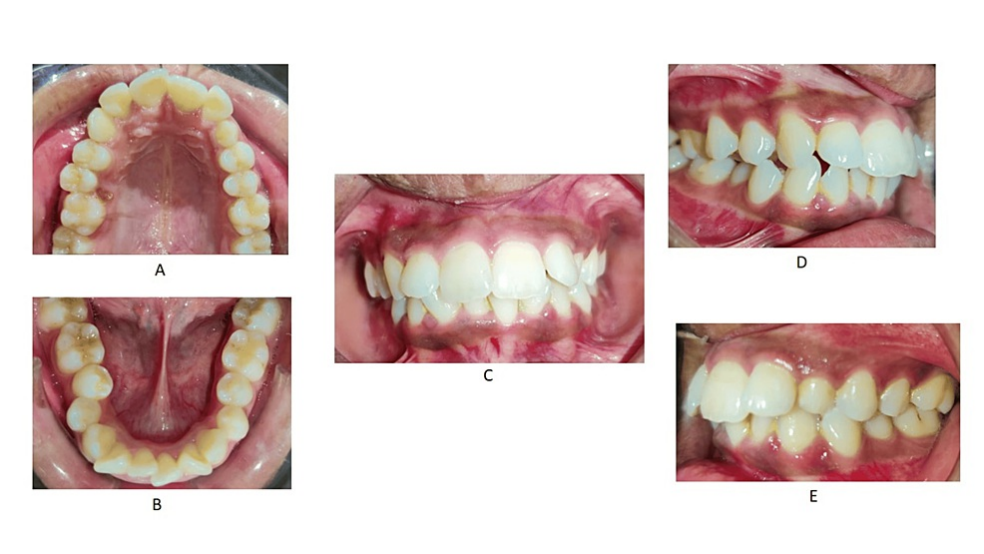
Pre-treatment intra-oral photographs (A) Maxillary arch, (B) mandibular arch, (C) anteriors in occlusion, (D) right molars in occlusion, and (E) left molars in occlusion

A 24-year-old female presented to the Department of Orthodontics with the chief complaint of irregularly and forwardly placed teeth in the upper and lower anterior region along with an over-retained deciduous tooth in the upper left region. She had a leptoproscopic facial form with a convex profile and a non-consonant smile (Figure 1). Intra-oral examination revealed that all teeth until the second molar except the upper left permanent canine are present in both the arches with over-retained deciduous canine in the maxillary arch on the left side. The gingival health was satisfactory and an adequate zone of attached gingival was present. All the teeth were present in both the arches except the upper left canine and third molars. Molars were in class I relationship. 4mm overjet and 3mm overbite were also seen (Figure 2). On the functional examination, the patient showed normal speech pattern, oro-nasal breathing, and a typical swallowing pattern. The path of closure of the mandible was normal without any deviation and no other associated signs or symptoms of temporomandibular disorder (TMD).

**Figure 3:**
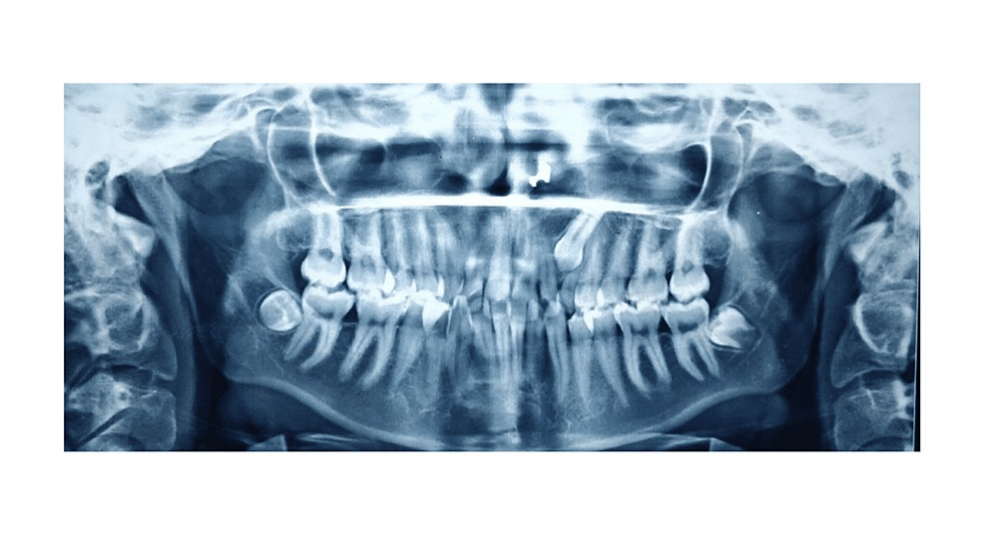
Pre-treatment OPG (orthopantomogram)

A panoramic examination revealed an impacted maxillary canine in the left upper quadrant (Figure 3). Cephalometric analysis revealed that the patient was in Cervical vertebrae maturation index stage VI (maturation) and had Class II skeletal bases, a vertical growth pattern. There was proclination of the upper and lower incisors. 

**Figure 4:**
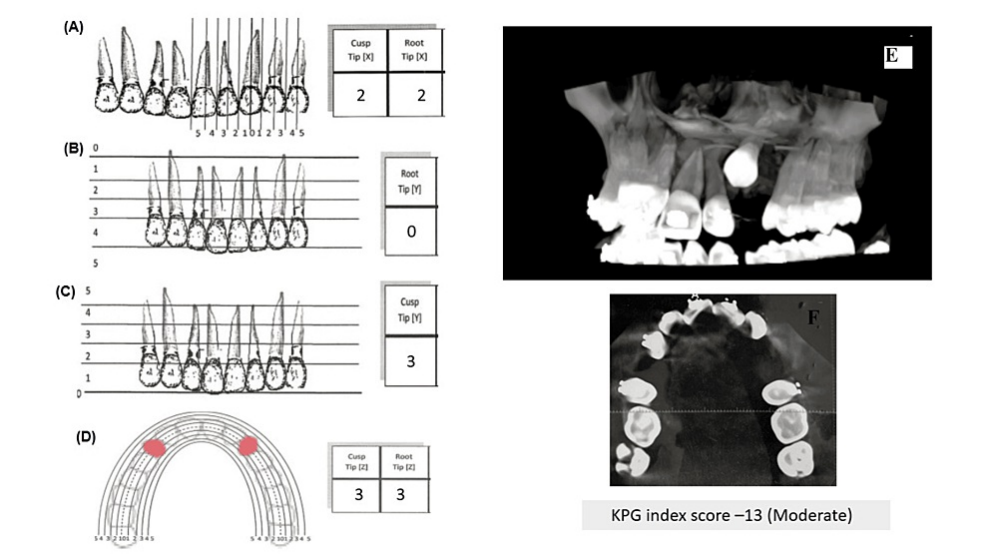
CBCT showing buccally impacted 23 and KPG index for difficulty index (A) Anterior-posterior dimension (X) for both cusp tip and root tip; frontal view; (B) vertical dimension (Y) for root tip; frontal view; (C) vertical dimension (Y) for cusp tip; frontal view; (D) deviation from the occlusal arch (Z); axial view; (E) CBCT showing the left maxillary quadrant; (F) CBCT showing the transverse maxillary occlusal view Source: Reference [6] CBCT: Cone beam computed tomography

 Cone beam computed tomography (CBCT) views of the left upper quadrant also show the impacted canine (Figure 4).

**Figure 5:**
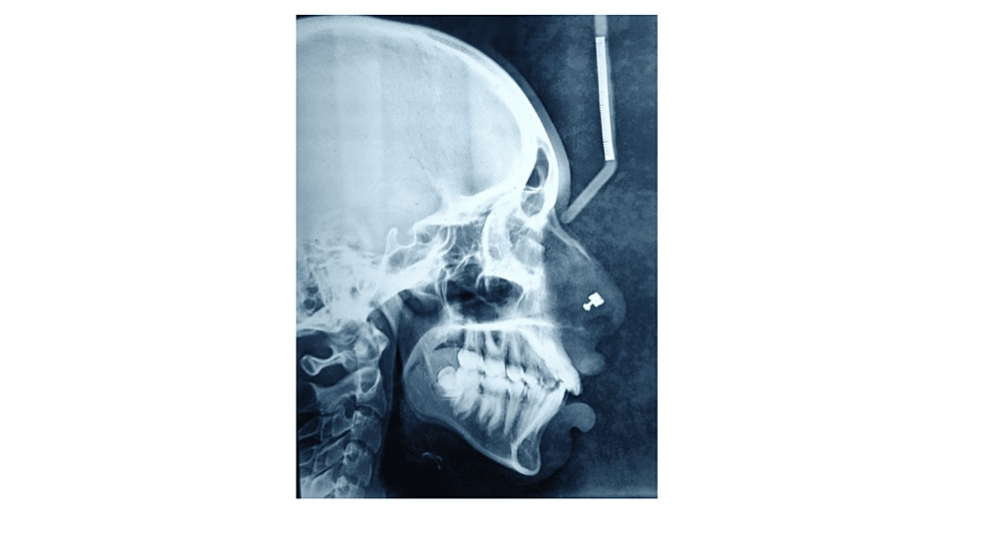
Pre-treatment lateral cephalogram

 The soft tissue analysis revealed a decreased nasolabial angle and shallow mento-labial sulcus (Figure 5).

The objective was to achieve class I canine relation bilaterally, satisfactory occlusion, closure of existing spaces, ideal overjet, overbite, and coincident midlines. This was done by proper alignment of the teeth, bringing the impacted canine into the arch, and correcting the proclined upper and lower incisors. The ideal facial esthetics and balance, together with a concordant smile, were desired.

To treat the case as critical anchorage in the upper and in the lower arch, the case was started with a pre-adjusted edgewise appliance (MBT 0.022” slot). Closed eruption of 23 followed by retraction of anterior teeth using loop mechanics was done.

**Figure 6:**
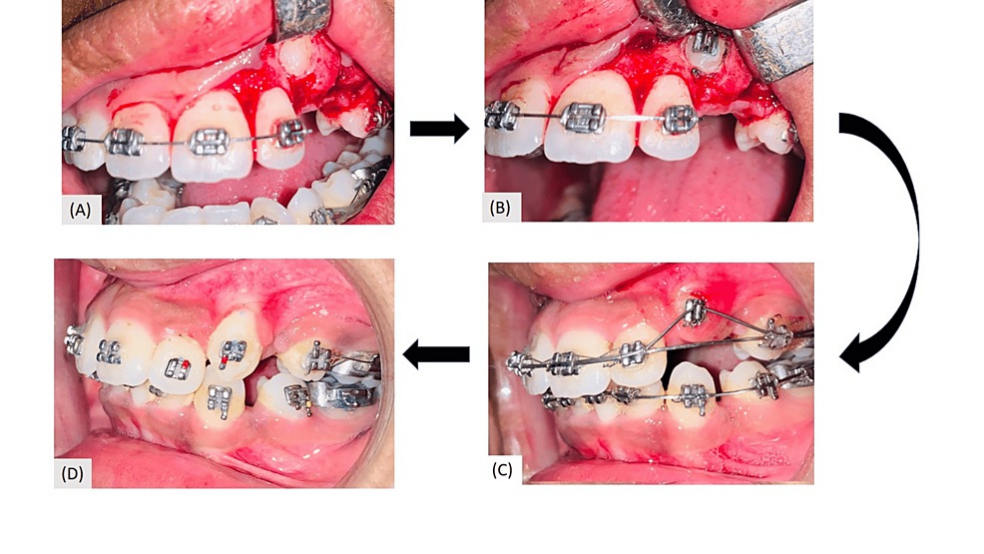
Surgical exposure and closed eruption of 23 (A) surgical exposure of 23; (B) bonding of Begg's bracket with 23; (C) piggyback with 0.012 NiTi wire; (D) attachment of inverted canine bracket after traction of 23

Therapeutic extraction of 14,24,34,44 and 63 as a part of the treatment protocol was done. As the case to be treated under critical anchorage, transpalatal arch (TPA) with maxillary and lingual arch with mandibular arch was given. Intraoral strap-up was done in both arches. Alignment was started with 0.016” Nickel-titanium wire in both the maxillary arch and mandibular arch and continued till 0.017×0.025’’ SS wire and the duration of alignment was four months. Canine exposure with full thickness mucoperiosteal flap was done in the department of Oral Surgery and a Begg’s bracket was bonded with 23 followed by piggyback using 0.012” Nickel Titanium wire (base arch wire was 0.017×0.025’’ SS wire) and after four months of traction, the canine was brought into occlusion using the closed eruption technique (Figure 6).

**Figure 7:**
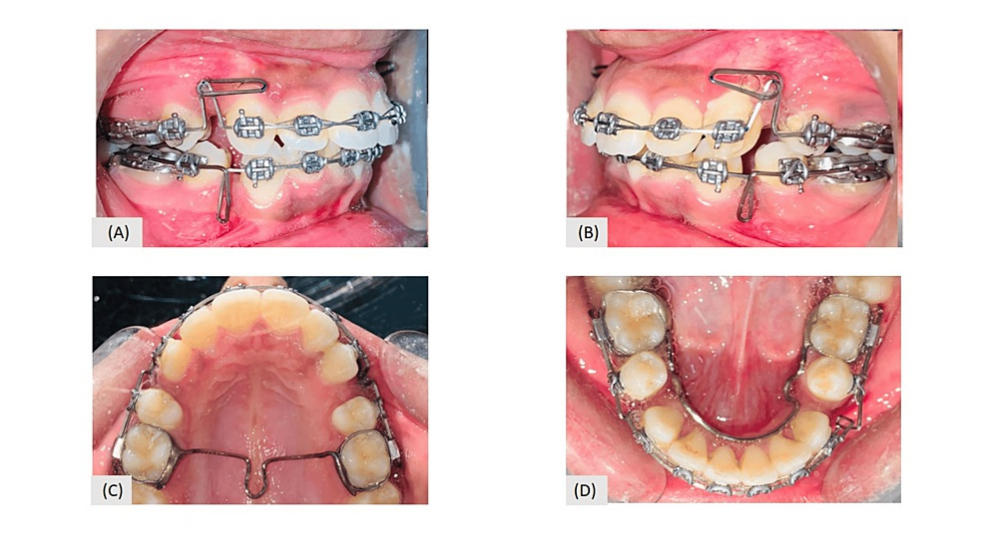
Retraction with loop mechanics (A) Opus loop in maxillary and Reverse loop in mandibular arch (right view); (B) opus loop in maxillary and Reverse loop in mandibular arch (left view); (C) maxillary occlusal view with loop; (D) mandibular occlusal view with loop

The retraction was started with an opus loop in the upper and a reverse loop in the lower arch with 0.017×0.025’’ Titanium molybdenum archwire (TMA) (Figure 7). After retraction finishing bends were also incorporated using 0.019×0.025’’ TMA wire for first and second order correction. The total duration of treatment was 1.5 years. 

**Figure 8:**
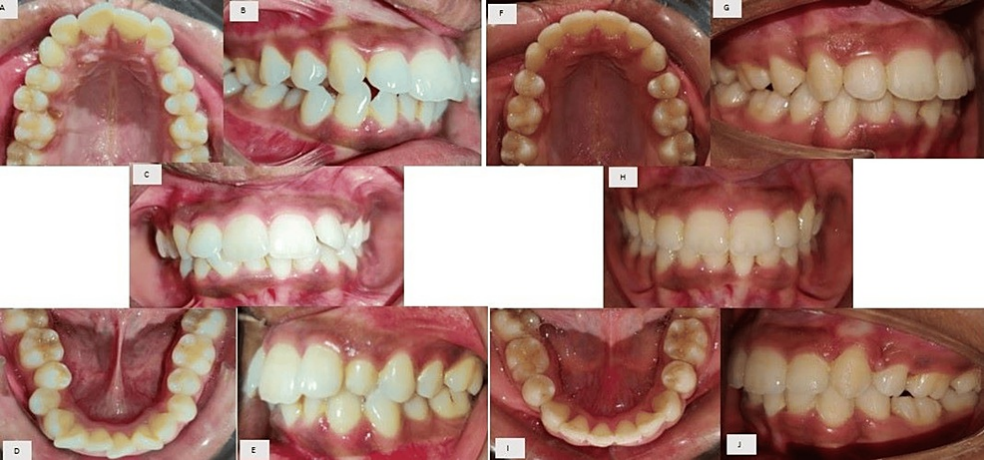
Comparison of pre and post-treatment intraoral photographs: Pre-treatment (A) Maxillary arch, (B) right molars in occlusion, (C) anteriors in occlusion, (D) mandibular arch, (E) left molars in occlusion; Post-treatment: (F) maxillary arch, (G) right molars in occlusion, (H) anteriors in occlusion, (I) mandibular arch, and (J) left molars in occlusion

**Figure 9:**
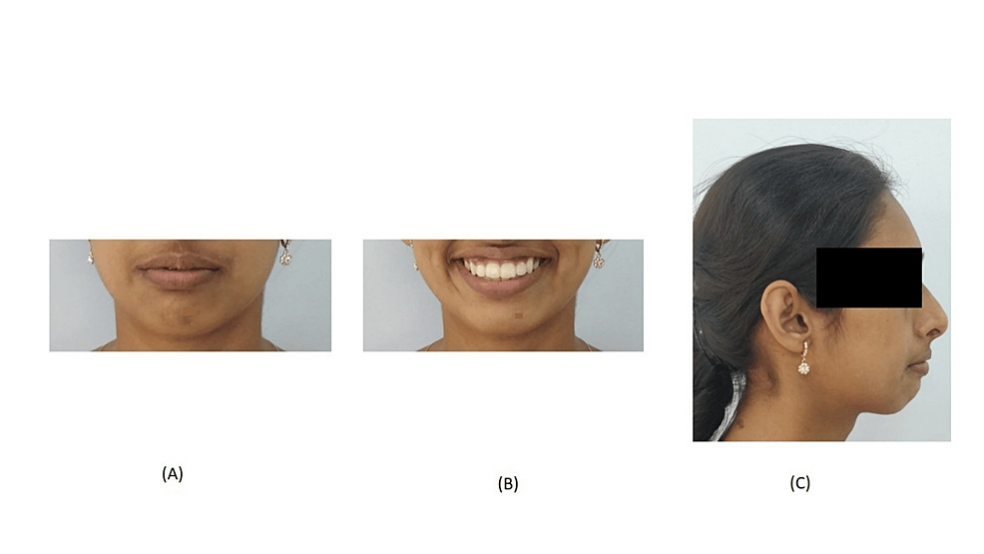
Post-treatment extraoral photographs (A) Frontal; (B) smiling; (C) profile

**Figure 10:**
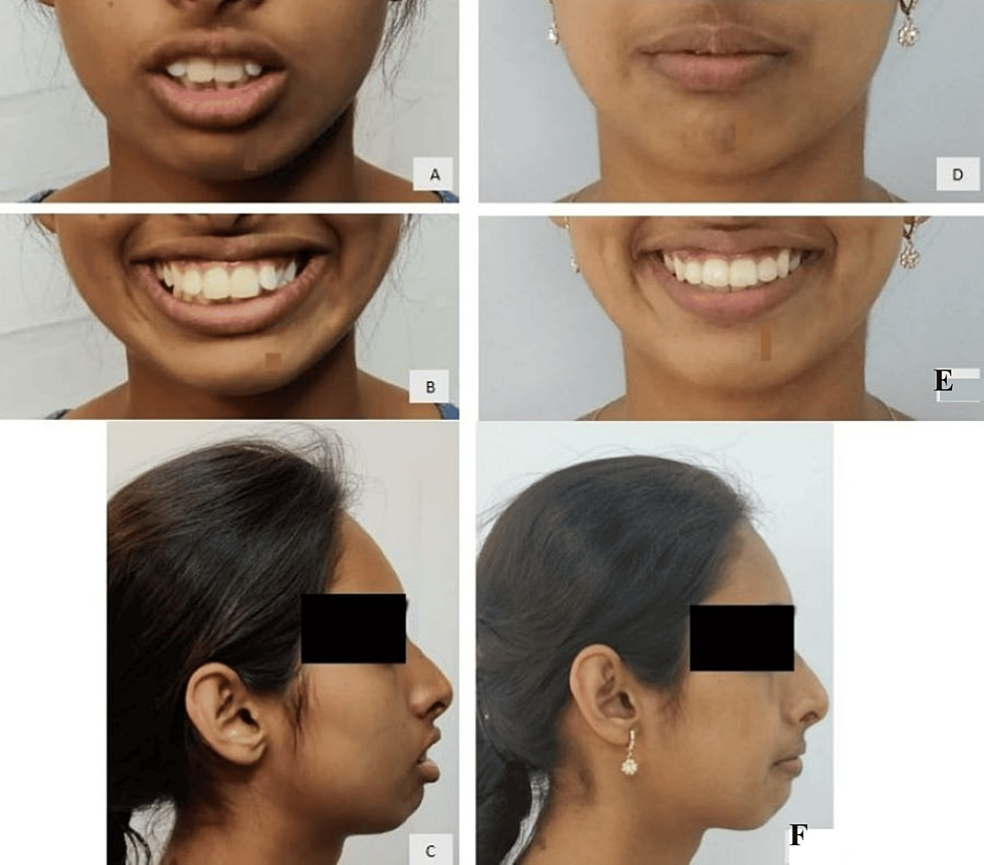
Comparison of pre- and post-extraoral photographs: Pre-treatment: (A) Frontal, (B) smiling, and (C) profile; post-treatment: (D) Frontal, (E) smiling, and (F) profile

**Figure 11:**
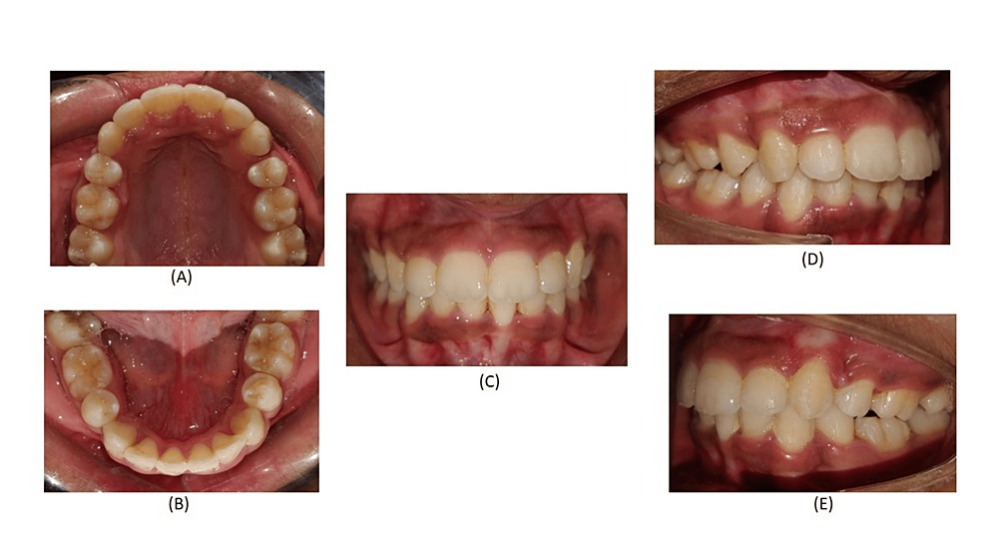
Post-treatment intra-oral photographs (A) Maxillary arch; (B) mandibular arch; (C) anteriors in occlusion; (D) right molars in occlusion; (E) left molars in occlusion

**Figure 12:**
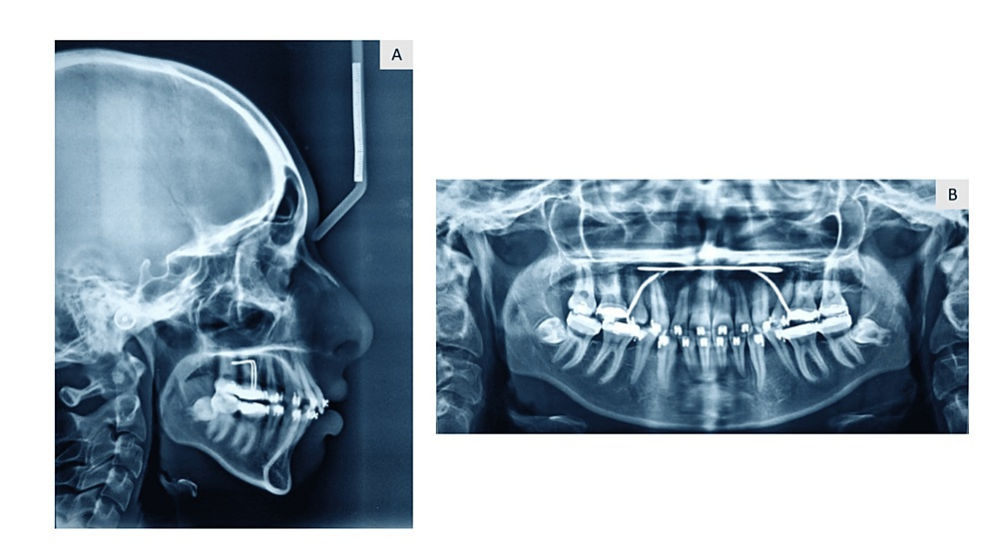
Post-treatment radiographs before settling of occlusion. (A) Lateral cephalogram and (B) OPG (orthopantomogram)

**Figure 13:**
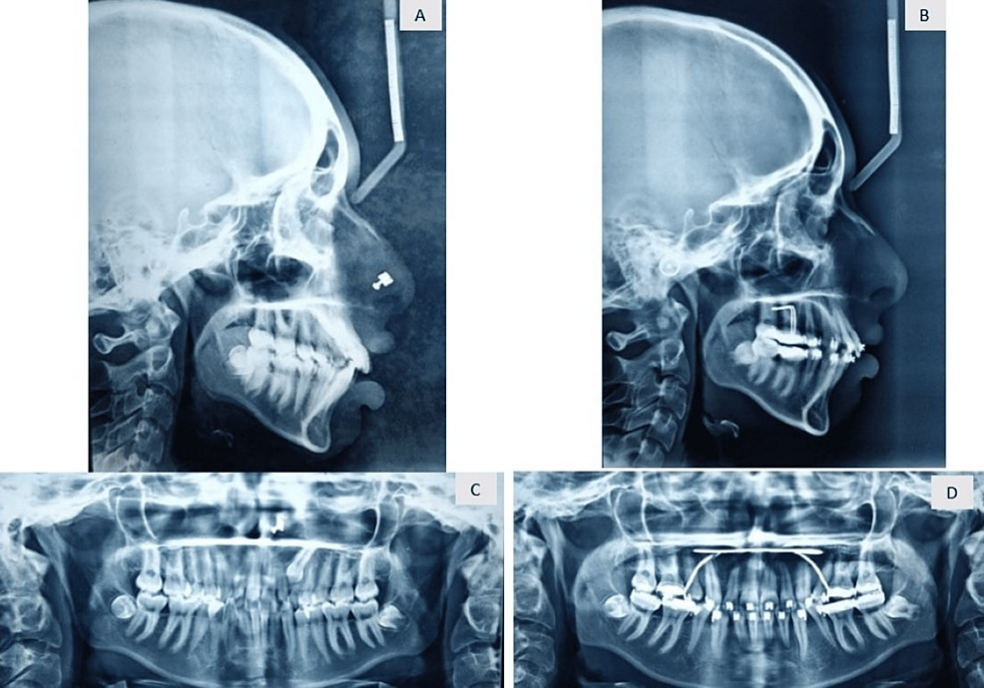
Comparison of pre- and post-treatment radiographs: pre-treatment: (A) OPG and (C) lateral cepahlogram and post-treatment: (B) OPG and (D) lateral cepahlogram OPG: Orthopantomogram

After the completion of the case, the patient’s chief complaint of irregularly placed and proclined anterior teeth was addressed (Figure 8). A consonant smile is obtained (Figure 9). Acceptable lip competency is achieved (Figure 10A). Retraction of the upper anterior is achieved. The impacted canine was brought into the arch (Figure 6). Class I canine relations were obtained bilaterally. Class I molar relations are maintained on the right and left side with ideal overjet and overbite along with optimal facial aesthetics and balance (Figure 11). Post-treatment radiographs show erupted maxillary upper left canine (23), class I molar relation and correction of proclination of both upper and lower incisors (Figure 12). Pre- and post-treatment cephalometric radiograph (Figure 13) values also show significant changes specifically in proclination of upper and lower anteriors (Table 1).

**Table 1:** Pre and post-treatment cephalometric values SNA: Sella nasion point A; SNB: Sella nasion point B; ANB: Point A nasion point B; FMA: Frankfort mandibular plane angle; UI: upper incisor; LI: lower incisor; IMPA: incisor mandibular plane angle; E: esthetic line

Variables	Mean	Pre-treatment	Post-treatment	Difference
Maxilla to cranium				
SNA (degree)	82±2	81	79	2
Mandible to cranium				
SNB (degree)	80±2	75	74	1
Maxilla to mandible				
ANB (degree)	2±2	6	5	1
Wits(mm)	0	4	1.6	2.4
Vertical relationship				
Y-axis angle (degree)	53–66	59	5	0
Facial axis angle (degree)	90	92	83	9
FMA angle (degree)	25	31	31	0
Occlusal to SN (degree)	23	15	19	4
Maxillary dental				
UI to NA (angle)	22	30	19	11
UI to NA (mm)	4	7	3	4
UI to SN (angle)	102±2	112	100	12
Mandibular dental				
LI to NB (angle)	25	32	27	27
LI to NB (mm)	4	8	6.9	1.1
IMPA (angle)	90±5	93	92	1
Maxilla to mandible (dental)				
UI to LI (angle)	130	115	123	8
Soft tissue analysis				
Nasomental angle	120-132	121	119	2
Nasolabial angle (degree)	102±4	92	94	2
Upper lip prominence (mm)	1-2	3	1	2
Lower lip prominence (mm)	1	6	-2	4
E-line (upper lip/lower lip)(in mm)	-4/-2	-3/5	-1/2	-2/3
Soft tissue chin thickness (mm)	10-12	10	8	2

## Discussion

The reported incidence of impacted maxillary canines in the literature, ranging from 0.8% to 2.8%, highlights the significance of this dental condition [1]. Although the exact etiology of canine impaction remains elusive, several factors have been proposed as potential contributors. Among them, the intricate path that the canine follows during its eruption process and the extended duration of its development have been recognized as significant influences on impaction [2,3]. Genetic or familial predisposition has been suggested as another potential cause of maxillary canine impaction [4]. However, in the present case, no familial background was detected, underscoring the complex and multifactorial nature of this condition. It is essential to acknowledge that canine impaction can occur sporadically and may not always exhibit clear familial patterns [5,6]. While the literature contains numerous case reports of successful treatments for impacted canines, this case falls into a moderate type of impaction (Figure 4) according to the KPG index (as done by San Martin et al.) along with loop mechanics used for retraction [7].

The impact of an impacted or unerupted tooth on both esthetics and function is undeniable. Particularly in cases where the affected tooth is located in the anterior region, the need for proper alignment within the dental arch is often paramount [8,9]. Canine impactions, characterized by their severity, typically necessitate surgical intervention. This involves a meticulous process of buccal flap reflection, bone removal overlying the impacted tooth to facilitate attachment placement, and subsequent orthodontic traction [10]. The success of such procedures hinges on the close collaboration between oral surgeons and orthodontists, whose combined expertise ensures not only optimal results but also a favorable long-term prognosis [11,12]. The integration of surgical orthodontic techniques into clinical practice has ushered in a new era of treatment for impacted canines [13]. Creating adequate space within the dental arch to accommodate the impacted teeth and subsequently performing surgical exposure, followed by mechanical traction, have become well-established protocols in the management of canine impaction [14]. The permanent canine is the most important unit of dentition as it is called as cornerstone of the oral cavity which provides not only a balanced smile and canine guidance but also maintains inter-canine width and prevents from collapse of the arch. Post-treatment, although the class I canine with ideal overjet and the overbite was achieved which is important for functional occlusion along with functionally stable cusp-embrasure, there was some space distal to the upper right canine.

## Conclusions

In conclusion, the incidence and etiology of impacted maxillary canines underscore the importance of early diagnosis and comprehensive treatment planning. The complex interplay of factors contributing to impaction necessitates a tailored approach for each patient. Collaborative efforts between surgeons and orthodontists, along with the utilization of surgical-orthodontic techniques, have revolutionized the management of impacted canines, ensuring improved aesthetics, function, and long-term oral health for affected individuals.
